# Do demographic factors and a health-promoting lifestyle influence the self-rated health of college nursing students?

**DOI:** 10.1186/s12912-018-0322-y

**Published:** 2018-11-29

**Authors:** Susan Ka Yee CHOW, Kin-Man LAM, Shih-Hung LIE, Ka-Chun MAK, Ka-Chun MONG, Chun-Man SO, Wai-Yip YUEN

**Affiliations:** School of Nursing, Tung Wah College, 31 Wylie Road, Homantin, Kowloon, Hong Kong

**Keywords:** Self-rated health, Health-promoting lifestyle, Nursing students

## Abstract

**Background:**

To adopt a healthy lifestyle is considered an essential component of nursing education. Self-rated health is a subjective assessment of health status and is consistent with objective health status. Previous studies have shown an association between self-rated health and engagement in a healthy lifestyle. Nursing students need to feel good about their subjective health status and to be able to adopt health improvements in their lifestyle before attempting to disseminate health messages to clients. The aims of this study were to compare the difference in self-rated health and health promotion lifestyle profile between senior and junior nursing students, describe correlations between self-rated health and health promotion lifestyle profile, and identify the predictors of self-rated health.

**Methods:**

A cross-sectional descriptive survey was adopted. The study sample consisted of 314 junior and senior year nursing students from a tertiary institution. The self-reported questionnaire consisted of a single-item question to examine their self-rated health. The Health Promoting Lifestyle Profile-II: Chinese version short form (HPLP-IICR) was used to investigate the health-promoting lifestyles of the students. Descriptive statistics, Mann-Whitney U test, Chi-square test, Fisher’s exact test, Spearman’s correlation, and ordinal logistic regression were used to analyze the data.

**Results:**

The median scores for self-rated health were 3 (Mean 3.26, IQR 3–4) and 3 (Mean 3.19, IQR 3–4) out of 5 for Year 2 and Year 5 students, respectively, with no significant difference between the two groups. The two groups of students showed no significant differences in overall score and in most subscales of the HPLP-IICR. An ordinal logistic regression showed that those students with higher health management score (OR: 1.12, 95% CI: 1.04–1.21) and who had experienced no family conflicts in the recent month than having family conflict (OR: 1.64, 95% CI: 1.01–2.66) were more likely to have higher self-rated health.

**Conclusion:**

Nursing education and clinical practice can undoubtedly increase the health knowledge of students, but may not lead to changes in actual health-promoting behaviours. Students with a higher health management score and no family conflicts are more likely to give a positive appraisal of their health status.

**Electronic supplementary material:**

The online version of this article (10.1186/s12912-018-0322-y) contains supplementary material, which is available to authorized users.

## Background

Healthcare professionals are role models in introducing health-promoting lifestyles to patients as well as to the public, and it has been shown that their own behaviours can influence how they counsel patients [1]. People leading unhealthy lifestyles not only contribute to high morbidity and reduced productivity in society, but can also be an economic burden on society [2]. Health promotions are largely concerned with the behaviour and lifestyle of individuals, as well with as the physical and social environment. Nevertheless, the main strategy is to promote a lifestyle conducive to health and to keep preventable conditions from developing [3]. Studies have shown an association between lifestyle factors such as alcohol use, smoking, and dietary habits and the incidence of cardiovascular diseases and diabetes, while the practice of safe sex has been related to a decline in the prevalence of HIV [4, 5]. A systematic review conducted in 2010 found empirical evidence that sedentary behaviours during childhood and adolescence, such as the excessive viewing of television and playing of electronic games and a lack of engagement in physical activities, are likely to shape an individual’s adult life [6]. In their systematic review and meta-analysis, Loef and Walach [7] concluded that the adoption of a combination of healthy behaviours, including refraining from smoking, engaging in regular exercise, maintaining an optimal body weight, and drinking less alcohol, was associated with a 66% reduction in mortality, resulting in a significant reduction in healthcare costs worldwide.

### Self-rated health and health

Self-rated health (SRH), also known as self-perceived health or self-assessed health, is a subjective assessment of health status and functional decline and is consistent with objective health status. The measurement scale for SRH has been widely used as a global measure of general health status in the public health arena [8]. In the clinical sector, a single general question on self-rated health is a strong and consistent predictor of mortality. Those patients with ‘poor’ self-rated health had twice the mortality risk of their counterparts with excellent self-rated health [9]. SRH is also a significant predictor of most chronic diseases found among the US population in late midlife, such as arthritis, lung disease, stroke, and coronary heart diseases, but not cancer [10]. SRH is not only an effective and accurate predictor of physiological health, but also of emotional health; poor SRH has been observed among unemployed young people due to the long-term negative emotional impact resulting from job loss and low self-esteem [11].

The above studies have shown that health-promoting lifestyles or self-rated health are eloquent predictors of well-being in both the public health and clinical arenas. For the younger population, a Korean study illustrated that spiritual growth, physical exercise, and stress management were related factors in university students’ perceptions of their health status [12]. An investigation on health behaviours, self-rated health, and quality of life was conducted among freshmen in a Swedish university. The results demonstrated that male students engaged in unhealthy lifestyles more than had been expected, while the students’ self-perceived quality of life was more strongly related to their self-rated psychological health than to their physical health. A local study in Hong Kong examined the association between self-rated health and adolescent drinking; the results revealed that suboptimal self-rated health was significantly associated with the drinking of alcohol [13].

Nursing students are a future generation of educated, motivated people with the professional knowledge to be role models on healthy lifestyles. They need to feel good about their subjective health status and to be able to adopt health improvements in their lifestyle before attempting to disseminate health messages to clients. A recent local study addressed possible potential barriers to the adoption of healthy lifestyles by nursing students, including a heavy academic workload and physical fatigue after a clinical practicum [14]. Although self-rated health is a subjective reflection of health status while the adoption of a healthy lifestyle is closely associated with objective health outcomes, the relationship between the two remains undetermined. Moreover, thus far there has been no study on the self-rated health and lifestyle profiles of junior and senior nursing students and on the predictors of self-rated health in relation to demographic data and healthy lifestyles. Therefore, the aims of this study were to compare the difference in self-rated health and health promotion lifestyle profile between senior and junior nursing students, describe correlations between self-rated health and health promotion lifestyle profile, and identify the predictors of self-rated health.

## Methods

### Study design

A cross-sectional descriptive survey was adopted in the design.

### Study setting and participants

The study was conducted in early 2018 in the School of Nursing of a tertiary institution in Hong Kong. Consecutive sampling was employed to invite all eligible students to participate. Consecutive sampling is a non-probability sampling technique where the researcher picks a group of people to participate the study [15]. The eligible participants were all full-time Year 2 and Year 5 bachelor’s degree nursing students representing junior and senior year students, respectively, and numbering around 450. Part-time and sub-degree nursing students were excluded from the study.

The primary outcome of the study was to compare the differences in self-rated health and health promotion lifestyle profile between senior and junior nursing students. As there was no previous study describing the correlations between self-rated health and health promotion lifestyle profile, and identify the predictors of self-rated health, the sample size calculation was based on a previous study on barriers hindering nursing students from adopting a health-promoting lifestyle [14]. The difference in the overall score in health promoting lifestyle among the Year 1 and Year 4 nursing students was used to determine the effect size between them. The effect size was estimated based the difference between two independent means of t-test divided by Standard Deviation. With an effect size of 0.298, a significance level of 0.05, a power of 0.8, and allocation ratio N2/N1 of 1, the minimum sample required was 356 subjects, with 178 in each group. G*Power was used to calculate the size of the sample. The software was designed as a stand-alone analysis programme for a number of statistical tests and has been widely used in the social, behavioural, and biomedical sciences [16].

### Instruments

The questionnaire for collecting data consisted of three sections. Section one was comprised of 11 self-developed questions on demographic characteristics, including on gender, year of study, religious beliefs, family relationships, and so on. Section two consisted of a single question on general self-rated health (SRH), answered using a 5-point Likert scale (1 = poor, 2 = not so good, 3 = average, 4 = good, 5 = excellent). The predictive ability of the scale was compared with that of the Diagnostic Cost-Related Groups/Hierarchal Condition Categories Relative-Risk Score, the Short Form-12, and the Seattle Index of Comorbidity. The single-item scale was able to predict future health expenditures as well as other complex health models [17]. A study showed evidence of the test-retest reliability of the single-item measure on general health status, with an Intraclass Correlation Coefficient of 0.86 [18].

The focus in section three was on investigating health-promoting lifestyles using questions adopted from the Health Promoting Lifestyle Profile-II: Chinese version short form (HPLP-IICR). The original Health Promoting Lifestyle Profile was developed by Walker [19] in 1987 and consists of 48 items. It has been translated into different languages and is used in various countries [20, 21]. The author further revised the scale, resulting in the 52-item HPLP II. The short form of the Chinese version (HPLP-IICR), a 30-item scale, was developed in 2010 and has proven to be as valid and reliable as the original version [22]. The results demonstrated the five-factor structure of the new Chinese version, namely ‘Spiritual growth’ (6 items), ‘Physical activity’ (6 items), ‘Health management’ (9 items), ‘Nutrition’ (4 items), and ‘Health responsibility’ (5 items). The items under ‘Health management’ focus on interpersonal relationships and activities to manage health, including stress management. The respondents were asked to answer the questions based on their health-promoting behaviours on a 4-point Likert scale from 1 = never to 4 = routinely. The score of the scale ranged from 30 to 120, with a higher score indicating a better health-promoting lifestyle. To determine its criterion-related validity, the HPLP-IICR was assessed by correlating it with the WHOQOL-BREF. Those participants who obtained higher scores in the HPLP_IICR had statistically significantly higher scores on quality of life as well as on perceived overall health status. The reliability of the scale was investigated through exploratory factor analysis, item analysis, and internal consistency. The Cronbach’s alpha for the entire scale was 0.90, while that for the subscales ranged from 0.69–0.87. The results explain the strong psychometrics of the HPLP-IICR for the Mandarin-speaking population. In this study, the Cronbach’s alpha of the entire scale was 0.89, the subscale for spiritual growth = 0.84, physical activities = 0.83, health management = 0.79, nutrition = 0.68 and health responsibility = 0.74. The scale was considered having acceptable internal consistency as the Cronbach’s alpha coefficients were between 0.68 and 0.90 for majority of the subscales as stated by Portney and Watkins [23].

To ensure the cultural relevance of the two scales to Hong Kong students, a content validity test was conducted prior to the collecting of data. Three academic staff members of a university were invited to determine the representativeness and relevance of all items in relation to the aims of the study. They deemed all of the items to be acceptable and relevant. The Content Validity Index of the scale was 1.0. Two phrases in the HPLP-IICR had to be revised to improve the cultural relevance and readability of the questions. An evaluation of test-retest reliability was conducted to determine the stability of the two scales subsequent to the running of a content validity test. Twenty university students were invited to complete the same questionnaire twice, with a two-week interval. The Intraclass Correlation Coefficient was 0.93 for SRH and 0.85 for the HPLP-IICR. The results indicated that the scale had good reliability, as stated by Portney and Watkins [23]. In the above validation exercise, the participants spent about 15–20 min completing the entire questionnaire (Additional file 1).

### Data collection

The data were collected in early 2018. Approval to distribute the questionnaires during class was sought from the teachers. All data were collected through self-administered questionnaires completed by the two groups of students. The students who consented to participate completed the questionnaires on the spot. Three hundred and eighty questionnaires distributed and 325 questionnaires were returned, for a response rate of 85.5%.

### Ethical considerations

The Human Research Ethics Committee of School Research Committee, School of Nursing, Tung Wah College granted their permission to implement the study. The Reference Number is: NUR/SRC/20171220/032. Informed consent was obtained from all of the nursing students who participated in the study.

### Data analysis

All statistical analyses were performed using SPSS version 23 (SPSS Inc., Chicago, IL., USA) and Microsoft Excel. Descriptive statistics (median, interquartile range, range, and percentages) were used to describe the demographic variables. Pearson Chi-square test or a Fisher’s Exact test was to examine the associations of the categorical variables between junior and senior students. A Shapiro-Wilk test was used to examine the normality of the overall and subscale scores of the HPLP-IICR and SRH. The data are considered to be normally distributed when the significant value is greater than 0.05 [23]. The majority of the variables were not normally distributed for the sub-scales of the HPLP-IICR. A non-parametric test, the Mann-Whitney U test, was used to examine the differences between the junior and senior year students on SRH and the HPLP-IICR. Spearman’s rho correlations were used to investigate the correlations between SRH and the subscale scores of the HPLP-IICR. Ordinal logistic regression with the calculation of Odds Ratio and 95% Confidence Interval was used to further examine whether demographic factors and the HPLP-IICR are significant predictors of SRH. The decision for which demographic variables to be included in multivariate ordinal logistic regression model is no two or more independent variables that are highly correlated, or the independent variable is having a very small percentage of sample comparing with the total sample. The purpose of calculating the Odds Ratio and 95% Confidence Interval is to exponentiate the Parameter Estimates. The results of the ordinal logistic regression were extracted to Microsoft Excel, with a simple formula developed to calculate the Odds Ratio to determine which variable has a large effect on outcome, and the magnitude of the effects for that outcome [24]. The exact formula in Excel is “EXP (beta)” for Odds Ratio, “EXP (Lower Bound)” and “EXP (Upper Bound)” for calculation of 95% Confidence Interval [25]. A *p* value of < 0.05 was considered to be statistically significant in a two-tailed test.

## Results

Eleven questionnaires were discarded because the amount of missing data exceeded 10% of the total items. In the end, 314 valid questionnaires were available for analysis.

Among the 314 participants, 169 students were from Year 2 and 145 students were from Year 5. The majority of the participants were female (78.3%) and living with their family (96.8%). About 71.7% of the students reported having a part-time job and most of those worked from 8 to 16 h per week. About one-fourth of the students held religious beliefs and around one-third had experienced a conflict with their family in the past month (Table 1).

**Table 1:** Demographic characteristics and life habits of the participants

Variable	Year 2 (*n* = 169)	Year 5 (*n* = 145)	Total (*n* = 314)	Results of association *p*-value
	N (%)	N (%)	N (%)	
Gender				0.66^a^
Male	35 (20.7)	33 (22.8)	68 (21.7)	
Female	134 (79.3)	112 (77.2)	246 (78.3)	
Clinical practicum				< 0.0001^a^
No	169 (100)	0 (0)	169 (53.8)	
Yes	0 (0)	145 (100)	145 (46.2)	
Religious beliefs				0.047^a^
No	120 (71)	117 (80.7)	237 (75.5)	
Yes	49 (29)	28 (19.3)	77 (24.5)	
Engaged in a part-time job				0.02^a^
No	60 (35.5)	29 (20)	89 (28.3)	
Yes	109 (64.5)	116 (80)	225 (71.7)	
No. of hours of part-time work (*N* = 227)				0.163^a^
< 8 h	41 (36.9)	27 (23.3)	68 (30)	
8 to 16 h	47 (42.3)	58 (50)	105 (46.3)	
17 to 24 h	18 (16.2)	24 (20.7)	42 (18.5)	
> 24 h	3 (4.5)	7 (6)	10 (5.3)	
Number of days eating outside in a week				0.821^a^
1 to 2 days	14 (8.3)	8 (5.5)	22 (7)	
3 to 4 days	54 (32)	48 (33.1)	102 (32.5)	
5 to 6 days	85 (50.3)	75 (51.7)	160 (51)	
7 days	16 (9.5)	14 (9.7)	30 (9.6)	
Living with family				0.522^b^
No	4 (2.4)	6 (4.1)	10 (3.2)	
Yes	165 (97.6)	139 (95.9)	304 (96.8)	
Conflicts with family members in the past month				0.097^a^
No	122 (72.2)	92 (63.4)	214 (68.2)	
Yes	47 (27.8)	53 (36.6)	100 (31.8)	

^a^Pearson’s Chi-square test

^b^Fisher’s Exact test

For self-rated health, the median score was 3 (Mean 3.26, IQR 3–4) and the range was 1–5 for the junior students, while for the senior students the respective figures were 3 (Mean 3.19, IQR 3–4) and the range was 2–5. No significant differences between the two groups were observed from the results of the Mann-Whitney U test, with *p* = 0.281.

Table 2 showed no significant differences in the overall score and in the subscales for spiritual growth (*p* = 0.891), physical activities (*p* = 0.807), health management (*p* = 0.884), nutrition (*p* = 0.182) and the overall score of HPLP-IICR (*p* = 0.404) between the junior and senior nursing students. There was a significant difference in health responsibility (*p* = 0.029), with the senior students performing much better than the junior students.

**Table 2:** Comparisons of HPLP-IICR scores by year of study among the nursing students

Variables	Year 2 (*N* = 169)		Year 5 (*N* = 145)		p-value
	*Median (IQR)*	Range	*Median (IQR)*	Range	
Spiritual growth	2.83 (2.33–3.17)	1.33–4.00	2.83 (2.50–3.00)	1.50–3.83	0.891
Physical activities	2.17 (1.83–2.67)	1.00–4.00	2.17 (1.67–2.67)	1.00–4.00	0.807
Health management	2.67 (2.44–3.00)	1.00–3.89	2.67 (2.44–3.00)	1.56–3.67	0.884
Nutrition	2.60 (2.20–3.00)	1.00–4.40	2.40 (2.20–2.80)	1.20–3.60	0.182
Health responsibility	2.00 (1.75–2.50)	1.00–3.25	2.25 (2.00–2.75)	1.00–3.75	0.029
Total HPLP-IICR	2.50 (2.23–2.67)	1.33–3.67	2.50 (2.28–2.78)	1.40–3.37	0.404

Table 3 presented that the relationships between SRH and the various subscales of the HPLP-IICR among all nursing students were statistically significant, with low to moderate positive correlations between SRH and the HPLP-IICR subscales. Moderate positive correlations were found between such subscales of the HPLP-IICR as ‘Health Management’ and ‘Spiritual Growth’ (*r* = 0.58, *p* < 0.001), and ‘Nutrition’ and ‘Physical Activities’ (*r* = 0.41, *p* < 0.001).

**Table 3:** Correlations between SRH and the HPLP-IICR (N = 314)

Variables	1	2	3	4	5	6
1. SRH	–					
2. Spiritual growth	0.27***	–				
3. Physical activities	0.24***	0.31***	–			
4. Health management	0.34***	0.58***	0.33***	–		
5. Nutrition	0.23***	0.36***	0.41***	0.34***	–	
6. Health responsibility	0.20***	0.35***	0.35***	0.31***	0.36***	–

**p < 0.05 ***p < 0.001

Because only a very small percentage of students (6.4%) rated their health as ‘Poor’ or ‘Very Good’, the items in original 5-point Likert scale of ‘Poor’ and ‘Not so good’, and ‘Good’ and ‘Very good’ were merged. The new categories for SRH were re-grouped into ‘Not so good’, ‘Average’, and ‘Good’ as the three ordered categories for the dependent variable in an ordinal logistic regression for ease of analysis. Table 4 showed that students who had a higher health management score (OR: 1.12, 95% CI: 1.04–1.21) and who had experienced no family conflicts in the recent month (OR: 1.64, 95% CI: 1.01–2.66) were more likely to have higher self-rated health. It was marginally significant for students who are religious to have higher self-rated health (OR 1.67, 95% CI: 0.98–2.85).

**Table 4:** Ordinal Regression Analysis on the predictors of SRH

Variables	Odds Ratio (95% CI)	*p*-value
Spiritual growth	1.03 (0.94–1.13)	0.474
Physical activities	1.05 (0.98–1.13)	0.131
Health management	1.12 (1.04–1.21)	0.001
Nutrition	1.09 (0.98–1.20)	0.088
Health responsibility	1.08 (0.96–1.22)	0.161
Number of hours working in a part-time job/week	0.94 (0.77–1.16)	0.611
Number of days eating outside/week	1.11 (0.82–1.51)	0.468
Gender		
Male	1.40 (0.78–2.51)	0.249
Female	1.00 ^Ref^	
Study year		
Year 2	1.38 (0.87–2.19)	0.171
Year 5	1.00 ^Ref^	
Religion		
None	1.67 (0.98–2.85)	0.058
Having religious beliefs	1.00 ^Ref^	
Family conflict		
None	1.64 (1.01–2.66)	0.042
Have family conflict	1.00 ^Ref^	

Ref = reference group

## Discussion

Our study is the first of its kind to compare the self-rated health and health-promoting lifestyles of junior and senior year nursing students, as well as the relationship between SRH and health-promoting lifestyles, and to examine the predictors of SRH among nursing students in a tertiary institution.

In our study, all Year 5 students had completed the required clinical practicum before graduation, while the Year 2 students had not commenced their clinical practice. It was presumed that the final year nursing students would demonstrate significantly better health-promoting behaviours than their junior counterparts. Contrary to our expectations, there were no significant differences between the two groups in the subscale scores of the HPLP-IICR, with the exception of ‘Health Responsibility’. The specific items for ‘Health Responsibility’ included discussing one’s own health concerns with healthcare professionals, inquiring about self-care methods from healthcare professionals, seeking a second opinion from physicians, and taking the initiative to report symptoms of abnormalities in their body to a physician. Differences in education increased over time. The significant difference in ‘Health Responsibility’ suggests that the effects of nursing education and clinical practice are linked to greater health consciousness and proactiveness on the part of the senior nursing students. Furthermore, the experiences gained from the clinical practicum cannot be underestimated, as the clinical practicum provides students with opportunities to deliver health promotion information to clients. Under the guidance of their clinical teachers, students can engage in reflections during the process of giving care and apply the knowledge that they have acquired to improving their own health. Furthermore, nurses have been encouraging patients to monitor their symptoms and promptly report them to healthcare professionals to promote early detection and treatment. The above evidence supports the notion that senior nursing students are better able to maintain their health responsibilities and to respond appropriately to health concerns than junior nursing students. Our findings are similar to previous studies conducted in Hong Kong and Turkey [14, 26], and partially consistent with a study in Thailand [27], revealing that freshmen reported being worse than more senior students at health responsibility, nutrition, and stress management.

There were insignificant differences between the two groups of students in other subscales of the HPLP-IICR. With regard to the demographic data, there were significant differences between the junior and senior students in engagement in a part-time job and having religious beliefs. Most of the senior students held part-time jobs and were not religious. Although these students should be more health conscious about managing their lifestyle because of their nursing education, they considered it a luxury to engage in daily physical exercise and enjoy leisure activities, as they were juggling a heavy study load, participating in clinical practices, and a part-time job. According to Sossah [28], nursing students face various stressors, with fear over their clinical performance tending to be their greatest source of stress. Apart from this, the effort to maintain meaningful interpersonal relationships, the uncertainty over future career choices, and having an inadequate support network further aggravated their stress levels. Although there was conflicting evidence on the association between religious beliefs and stress, a systematic review showed that religion and spirituality can help people to deal with the aftermath of trauma and to nurture relationships with others [29]. Another previous study also identified a significant negative correlation between perceived stress and spiritual well-being in a group of community dwelling adults after a six-week spiritual intervention [30]. The above studies explain some of the connections between spirituality and stress relief. If the majority of the senior students hold a part-time job and are not religious, this could explain the high levels of stress associated with their relatively low scores on ‘Health Management’ and ‘Physical Activities’. As for the junior students, they face enormous challenges, such as the need to adapt to new teaching and learning methods in tertiary education and to develop effective and meaningful relationships with peers, which may contribute to negative health-promoting lifestyles. Mirghafourvand et al.’s study [31] supported the view that social support, self-efficacy and mother’s occupation are predictors of a health-promoting lifestyle among adolescent girls. Apart from providing support to nursing students to determine their study and career goals and to find a purpose in life, nurse educators should teach students about how to expand their social networks and about various stress management techniques to increase their self-efficacies. These techniques include mindfulness-based stress reduction, progressive muscle relaxation, cognitive behavioural therapy, and so on [32]. It is only through using multiple strategies that nursing students can be empowered to manage their lifestyle and make a fair appraisal of their well-being.

Our results revealed no significant difference in SRH among the two groups of students. The previous literature review showed that suboptimal self-rated health is associated with unhealthy lifestyle behaviours, such as inadequate physical exercise and poor stress management, in young population groups. Since both groups of students showed no significant differences in health-promoting lifestyle, the findings lend further support for the view that a healthy lifestyle can influence a person’s perception of their own health. This concurs with the findings of Molarius et al. [33] to support the notion that poor self-rated health is independently related to both psychosocial as well as lifestyle factors among men and women.

Regarding the correlations in the subscale scores of the HPLP-IICR, our results showed a moderate positive relationship between ‘Health Management’ and ‘Spiritual Growth’ among the nursing students. The items for ‘Health Management’ include interpersonal relationships and stress management, while the items for ‘Spiritual Growth’ include holding out hope for the future, having opportunities to face new challenges, and receiving support from a group of people who care. The above are subjective evaluations of the degree to which an individual feels fulfilled in terms of needs, goals, and life satisfaction. There is evidence showing that adolescents are facing a great deal of anxiety, and that having someone, such as a friend or adult, to consult would alleviate their depression and increase their level of satisfaction with their life at school [34]. Hui concluded that having an increased ability to cope with stress and adversity will result in higher life satisfaction and better academic performance [35]. As ‘Health Management’ implies interpersonal relationships and stress management, if students are able to manage their time well, maintain fulfilling relationships with other people, and find the time to relax daily, this will tend to lead to better self-care and to better self-rated health. According to Saravia and Chau [36], manageability is a description of whether a person is capable of coping with demanding situations and has the resources to do so. Being able to relax and manage good interpersonal relationships has emotional meaning that leads to a subjective appraisal of health. Comprehensive and well-coordinated stress management programmes that include ways to identify coping strategies and cultivate resilience should be included in the undergraduate nursing programme to avoid threats to mental well-being and to enhance the students’ determination to face new challenges. Likewise, time management programmes, including the assignment of leisure time, would not only provide students with opportunities to relax and get along with others but also help them to participate in enjoyable activities that would promote well-being and fulfilment in their lives [37].

The finding of a moderate positive correlation between nutrition and physical activities is supported by earlier studies indicating that adolescents with an optimal Body Mass Index and good nutritional status are more active and spend less time on sedentary activities. Their healthy life habits include the regular consumption of fruit and dairy products and regular engagement in physical activities [38, 39]. Most tertiary institutions in Hong Kong do not require nursing students to participate in mandatory sports programmes as extra-curricular activities. Perhaps because of this, changes in attitude regarding the importance of physical exercise have not occurred among the nursing students, despite the instillation of theoretical knowledge on the subject during their studies. It is therefore necessary for educators to develop comprehensive, continuous, and inclusive physical education sessions despite their busy teaching schedule. The use of rewards or incentives for nursing students who demonstrate the initiative to participate in sports activities should be considered to promote lifestyle changes in tertiary institutions.

To further examine the predictors of SRH, the final model showed that the two variables with a significant direct effect on SRH were having a higher score in ‘Health Management’ and experiencing no recent family conflicts. Our results agreed with those of two recent studies conducted in Japan and Egypt on the relationship between work-family conflict and poor SRH [40, 41]. These studies revealed that men and women who experienced high work-family conflict tended to perceive their health status as poor. Another study, which examined the role played by the family environment of university students, indicated that non-cohesion, conflict, and control play crucial roles in the occurrence of depressive symptoms [42]. Strong and harmonious family ties are crucial factors in a student’s well-being. According to recent statistics [43], the number of divorces has increased continuously in Hong Kong, with the figure for 2016 being more than double that in 1991. Cross-border marriages between individuals from Hong Kong and mainland China made up 34.7% of registered marriages in Hong Kong in 2016, and have become a significant component of Hong Kong marriages [44]. Divorce, single and separate parenting, remarriage, and the formation of step-families are having pervasive effects on family relationships, resulting in temporary or permanent disruptions and leading to an increase in conflicts among family members [45]. These family conflicts and misunderstandings can be particularly painful and often affect the health of family members, particularly their emotional and psychological health. One study showed that family dinners play a significant role in adolescent development and emotional well-being. The frequency with which an individual has dinner with family members is positively related to life satisfaction and mental health [46]. When there is good bonding in the family, family meals can contribute to the development of fewer symptoms of depression in adolescents [47]. In working with adolescents with emotional problems, other than to provide the usual counselling services, adolescents could be encouraged to have family meals, as these are associated with psychological well-being and considered an effective approach to enhancing self-rated health. However, family dinners are of little benefit if parent-child relationships are not strong.

### Limitations of this study

For the cross-sectional survey of participating nursing students, a self-completed questionnaire was used, making it hard to generalize the results to all nursing students. Further studies could be conducted with a random sample from different institutions that offer nursing education, recruited using a more comprehensive sampling technique. This study indicates that interviews can be used to triangulate the findings on health-promoting behaviours, which could lead to a better understanding of the relatively low scores on health-promoting behaviours that were found among the senior year nursing students. The statistical power of this study was 0.75, which was slightly lower than 0.80, there is a need to replicate the study with a larger sample to increase the power of the test.

## Conclusion

Despite its limitations, the present study provided information on the health-promoting behaviours and self-rated health of junior and senior year nursing students, and identified the predictors of self-rated health from demographic factors and subscales of the HPLP-IICR. Nursing education and clinical practice can undoubtedly increase the necessary health knowledge of nursing students, but may not change their attitudes and actual health-promoting behaviours. The correlations were determined between various dimensions of a health-promoting lifestyle, such as self-management and spiritual growth, nutrition, and physical activities. Students with a higher health management score and no family conflicts were more likely to give a positive appraisal of their health status. It is recommended that, through the use of multiple strategies, nursing students be empowered to manage their lifestyle and make a fair appraisal of their well-being.

## Additional file

Additional file 1: Questionnaire. The questionnaire consisted questions for personal demographics, self-rated health and Health Promoting Lifestyle Profile II. (DOCX 40 kb)

## Abbreviations

CI: Confidence interval
HPLP-IICR: Health promotion lifestyle profile-II: Chinese version short form
M: Mean
OR: Odds ratio
SD: Standard deviation
SRH: Self-rated health
