# Supplementary material for: Do demographic factors and a health-promoting lifestyle influence the self-rated health of college nursing students?
Source: BMC Nurs. 2018 Nov 29;17:50. doi: 10.1186/s12912-018-0322-y (PMC6267045; doi:10.1186/s12912-018-0322-y)
Supplement: Supplementary file 1 — Questionnaire. The questionnaire consisted questions for personal demographics, self-rated health and Health Promoting Lifestyle Profile II. (DOCX 40 kb) [file 12912_2018_322_MOESM1_ESM.docx]

**Section 1: Demographic information**

1. What is your gender?

1 Male 2 Female

2. Which year of study are you currently in?

1 Year 2 2 Year 5

3. Have you been to clinical practicum before?

1 No 2 Yes

4. Do you have any religion?

1 No 2 Yes

5. Do you work part-time?

1 No (Go to Q7) 2 Yes

6. How many hours a week for your part-time job?

1 Under 8 hours 2 8-16hours 3 17-24hours 4 >24hours

7. How much time you need to spend on travel each day?

1 <30 minutes 2 30-60 minutes

3 61-120 minutes 4 >120 minutes

8. On average, how many days you need to dine out?

1 1-2 days 2 3-4days 3 4-6days 4 7days

9. Are you living with your family?

1 No 2 Yes

10. Did you have family conflict in the past month?

1 No 2 Yes

11. Do you smoke?

1 No 2 Yes

**Section 2: Self-rated health**

1. How do you rate your current health?

Very Good

Good

Fair

Poor

Very Poor

Section 3: Health Promotion Lifestyle Profile

| Questions | Never | Sometimes | Often | Routinely |
| --- | --- | --- | --- | --- |
| 1. Discuss my health concerns with health professionals | 1 | 2 | 3 | 4 |
| 2. Follow a planned exercise programme | 1 | 2 | 3 | 4 |
| 3. Feel I am growing and changing in positive ways | 1 | 2 | 3 | 4 |
| 4. Praise other people easily for their achievements | 1 | 2 | 3 | 4 |
| 5. Exercise vigorously for 20 or more minutes at least 3 times a week | 1 | 2 | 3 | 4 |
| 6. Take some time for relaxation each day | 1 | 2 | 3 | 4 |
| 7. Believe that my life has purpose | 1 | 2 | 3 | 4 |
| 8. Maintain meaningful interpersonal relationship | 1 | 2 | 3 | 4 |
| 9. Take part in light to moderate physical activity | 1 | 2 | 3 | 4 |
| 10. Look forward to the future | 1 | 2 | 3 | 4 |
| 11. Eat 2-4 servings of fruits each day | 1 | 2 | 3 | 4 |
| 12. Get a second opinion when I question my health care provider’s advice | 1 | 2 | 3 | 4 |
| 13. Take part in leisure time physical activities | 1 | 2 | 3 | 4 |
| 14. Concentrate at pleasant thoughts at bedtime | 1 | 2 | 3 | 4 |
| 15. Find it easy to show concern, love and warmth to others | 1 | 2 | 3 | 4 |
| 16. Eat 3-5 servings of vegetables each day | 1 | 2 | 3 | 4 |
| 17. Discuss my health concerns with health professionals | 1 | 2 | 3 | 4 |
| 18. Do stretching exercises at least 3 times/week | 1 | 2 | 3 | 4 |
| 19. Work toward long term goal in my life | 1 | 2 | 3 | 4 |
| 20. Touch and am touched by people I care about | 1 | 2 | 3 | 4 |
| 21. Eat 2-3 servings of milk, yoghurt or cheese each day | 1 | 2 | 3 | 4 |
| 22. Get exercise during usual day activities | 1 | 2 | 3 | 4 |
| 23. Eat only 2-3 servings from meat, poultry, fish, dried beans, eggs and nut group each day | 1 | 2 | 3 | 4 |
| 24. Ask for information from health professionals about how to take good care of myself | 1 | 2 | 3 | 4 |
| 25. Practice relaxation or meditation for 15-20 minutes daily | 1 | 2 | 3 | 4 |
| 26. Am aware of what is important to me in my life | 1 | 2 | 3 | 4 |
| 27. Get support from a network of caring friends | 1 | 2 | 3 | 4 |
| 28. Pace myself to prevent tiredness | 1 | 2 | 3 | 4 |
| 29. Eat breakfast | 1 | 2 | 3 | 4 |
| 30. Expose myself to new experiences and challenges | 1 | 2 | 3 | 4 |
